# Convolutional Neural Network-Based Deep Learning Methods for Skeletal Growth Prediction in Dental Patients

**DOI:** 10.3390/jimaging10110278

**Published:** 2024-11-02

**Authors:** Miran Hikmat Mohammed, Zana Qadir Omer, Barham Bahroz Aziz, Jwan Fateh Abdulkareem, Trefa Mohammed Ali Mahmood, Fadil Abdullah Kareem, Dena Nadhim Mohammad

**Affiliations:** 1 Department of Basic Sciences, College of Dentistry, University of Sulaimani, Sulaimaniyah 46001, Iraq; miran.mohammed@univsul.edu.iq; 2 Department of POP, College of Dentistry, Hawler Medical University, Erbil 44001, Iraq; zana.qadir@hmu.edu.krd; 3 Department of Prosthodontics, College of Dentistry, University of Sulaimani, Sulaimaniyah 46001, Iraq; barham.aziz@univsul.edu.iq (B.B.A.); jwan.abdulkareem@univsul.edu.iq (J.F.A.); 4 Department of Orthodontics, College of Dentistry, University of Sulaimani, Sulaimaniyah 46001, Iraq; trefa.ali@univsul.edu.iq; 5 Department of Pedodontics and Community Oral Health, College of Dentistry, University of Sulaimani, Sulaimaniyah 46001, Iraq; 6 Department of Oral Diagnosis, College of Dentistry, University of Sulaimani, Sulaimaniyah 46001, Iraq; dena.mohammad@univsul.edu.iq

**Keywords:** artificial intelligence, multiclass classification, growth maturation prediction, lower 2nd molar

## Abstract

This study aimed to predict the skeletal growth maturation using convolutional neural network-based deep learning methods using cervical vertebral maturation and the lower 2nd molar calcification level so that skeletal maturation can be detected from orthopantomography using multiclass classification. About 1200 cephalometric radiographs and 1200 OPGs were selected from patients seeking treatment in dental centers. The level of skeletal maturation was detected by CNN using the multiclass classification method, and each image was identified as a cervical vertebral maturation index (CVMI); meanwhile, the chronological age was estimated from the level of the 2nd molar calcification. The model’s final result demonstrates a high degree of accuracy with which each stage and gender can be predicted. Cervical vertebral maturation reported high accuracy in males (98%), while females showed high accuracy of 2nd molar calcification. CNN multiclass classification is an accurate method to detect the level of maturation, whether from cervical maturation or the calcification of the lower 2nd molar, and the calcification level of the lower 2nd molar is a reliable method to trust in the growth level, so the traditional OPG is enough for this purpose.

## 1. Introduction

Artificial intelligence (AI) significantly enhances efficiency, accuracy, and treatment planning in dental treatment. It improves healthcare decision-making, therapy, and rehabilitation. Furthermore, it aids in the identification of dental and skeletal abnormalities by radiographic interpretation and assessing growth and development [1,2]. Estimation of craniofacial growth represents the main target of preventive and treatment programs in orthodontics [3].

Proper diagnosis of a patient’s tooth development and craniofacial growth significantly impacts treatment plans. Interestingly, facial and dental structures change over time. This helps orthodontists anticipate growth patterns and assess the potential impact of orthodontic interventions. It gives clues in decision making regarding the timing and nature of orthodontic treatments and results outcomes [4,5]. Understanding the development of teeth and craniofacial structures in this young age group forms the basis for intervention strategies that aim to reduce malocclusion within the population [6].

Various biological indicators predict the human growth stage, such as chronological age, dental development, sexual maturation, and skeletal age. Many researchers have found that skeletal maturity is closely related to craniofacial growth [7].

The cervical vertebrae can indirectly influence orthodontic treatment due to their connection to the craniofacial complex. The relationship between the cervical vertebrae and orthodontics is often considered when assessing skeletal maturity and growth patterns. Orthodontists may consider the growth status of the cervical vertebrae when planning treatments related to jaw relationships, tooth alignment, and overall facial harmony [8,9]. Determining the growth potential at this stage of development could enable early short-term interceptive orthodontic treatment with simple appliances that reduce the complexity or even bypass the need to use other expensive orthodontic interventions [10].

Previous studies indicated that lateral cephalometric radiographs could detect cervical vertebral maturation (CVM) level as a dependable factor for the circum-pubertal growth phase [9,11]. This knowledge supports preventive orthodontic measures by enabling more precise timing for orthodontic interventions [10].

Tooth development is often linked with skeletal maturity. Panoramic radiographic interpretation can provide information about the stage of dental development. Interceptive orthodontic treatment may be required to manage specific malocclusion during the mixed dentition phase, and root development influences such intervention [12,13].

The use of osseointegrated implants has been increasingly widespread in the adult population. The literature shows a certain lack of application of this technique in children. The bone growth and development must be well analyzed, and the pediatric dentist might suggest using this treatment option for oral rehabilitation when necessary [14].

Dental implant placement is one of the possible modes of rehabilitation in pediatric patients with conditions such as congenital partial anodontia and traumatic tooth loss. Systematic planning of treatment is required to achieve optimum esthetic and functional outcomes. Furthermore, growth assessment accompanied by alveolar bone evaluation is mandatory to plan implant treatment. For more significant outcomes of implant treatments, all surgical and orthodontic procedures should be initiated about a year before the implant placement. In children, the greater the physiologic harmony that can be created within the dentition, alveolar bone, and skeletal growth changes, the higher the chances of successful implant placement. In determining the optimal individual time point of implant insertion, the status of skeletal growth, the degree of hypodontia, and the extension of related psychological stress should be considered in addition to the status of a pediatric patient’s existing dentition and dental compliance [15].

Deep learning models lead to remarkable improvements in image processing and segmentation. It modified interpreting dental radiographs towards automatic diagnosis and treatment. It could perform dental structure segmentation, classification, and identification of dental diseases with significantly high accuracy [16].

A study done by Kafieh and Aghazadeh focuses on using CNNs to classify tooth maturity stages based on panoramic radiographs, highlighting the effectiveness of traditional imaging techniques for dental assessments. The CNN model demonstrated high accuracy in classifying different stages of tooth maturity. The study reported effective performance metrics, including precision, recall, and F1-score, indicating that the model could reliably differentiate between various maturity levels [17]. Although there was no study correlating the skeletal maturity with dental classification, this study aimed to predict the skeletal growth maturation by convolutional neural network-based deep learning methods using cervical vertebral maturation and the lower 2nd molar calcification level so that skeletal maturation can be detected from an orthopantomography (OPG) image. No previous study was done with the use of multiclass classification on image identification, marking a significant advancement in the application of AI to preventive measures in dentistry and orthodontics.

## 2. Materials and Methods

### 2.1. Registration for Study

The University of Sulaimani’s College of Dentistry’s ethics committee granted this study’s clearance (199 on 10 December 2023). The authors disclose that the study was carried out in adherence to the principles and guidelines of the Helsinki Declaration. Additionally, the participants provided informed consent to be included in the study.

### 2.2. Sample

**Figure 1:**
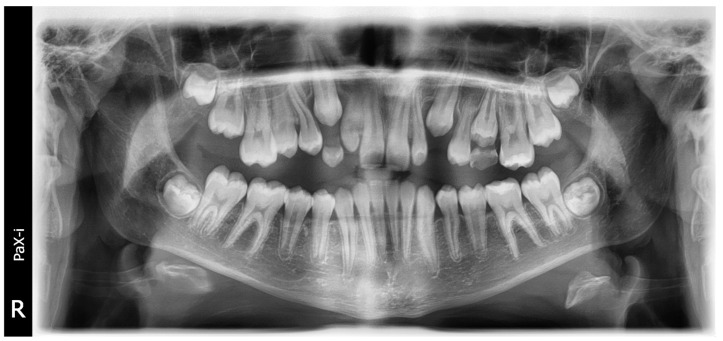
OPG of an 11.5-year-old female showed open apex of lower 2nd molars.

**Figure 2:**
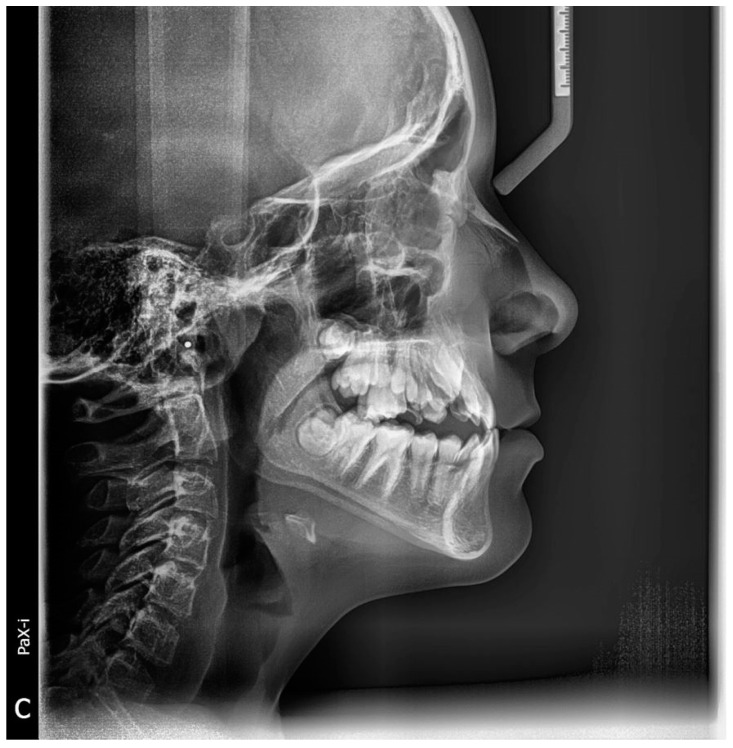
A cephalometric radiograph of the same patient revealed cervical vertebral maturation.

Two thousand and four hundred radiographic images (including 1200 cephalometric radiographs and 1200 OPGs as shown in Figure 1 and Figure 2) from different patients seeking dental treatment in private dental clinics were chosen with the ages ranged between 8 and 16 years, in which no previous orthodontic treatment or orthognathic surgery was performed and no medical history or medication interfered with the natural growth process. All the radiographic images were obtained by an experienced technician.

The X-ray machine used in the study is Vatech PaX-i3D (Vatech Company, Hwaseong-si, Republic of Korea), Model PHT6500 with a focal spot of 0.5 mm IEC 60336 and output of 90 Kv,10 mA, with total filtration of 2.8 mm AI. The software for OPG and cephalometric radiation and exposure time is adjusted by the machine automatically according to the age.

CVMI was evaluated by classifying cervical vertebra C_2_, C_3_, and C_4_ into six stages depending on their maturation patterns on a lateral cephalogram using a method given by Mcnamara, Baccetti, and Franchi, 2005 [18].

The analysis consisted of both cephalometric (quantitative) and visual (qualitative) appraisals of morphologic characteristics of the cervical vertebrae, revealing that statistically significant distinctions can be made between the stages of cervical vertebral maturation (Appendix A). Moreover, the mandibular right second molar was used (Figure 2). Tooth calcification was rated according to the index described by Demirjian et al. (Demirjian index DI; 1973), in which one of eight stages of calcification (A to H) was assigned to the tooth (Appendix B) [19].

### 2.3. Study Protocol

This work proposes a new method using AI techniques, especially a deep learning mechanism called CNN (convolutional neural network) that works on image classifications as binary and multiclass classifications. Binary classification works on distinguishing between two classes. While multiclass, it works on distinctive between more than two classes.

However, this paper is concerned with differentiating between multiple classes, which consist of 6 classes of cervical vertebrae maturation extracted from cephalometric radiography, which are named (CS1, CS2, CS3, CS4, CS5, CS6). Also, another prediction of lower 2nd molar calcification level according to Demirjian index DI; 1973 named in (D1, D2, D3, D4, D5, D6, D7), as the patients seeking orthodontic treatment aged mainly between 8 and 16 years old, so the first two categories (D1, D2) were not involved in this study.

Each section of classification prediction is operated in a separate process. And for each process, a CNN model is used with a different set of network architecture, consisting of the number of layers. In addition, later both results from the prediction were tested for correctness and accuracy, using different classification metrics, such as precision, recall, F1-score, confusion matrix, and accuracy as well. These metrics are implemented on architecture, cervical, and molar class predictions.

Moreover, these two models’ architecture of cervical and molar was implemented on genders, males and females. Also, the same classification metrics are implemented on both genders in each CNN model.

The strategies are started by collecting images of different patients with not less than 200 images for each class in molar OPG and cervical images. These images are separated into different groups and categories (molar, cervical, and genders for each one of them) and main classifications based on growth or ingrowth. Also, each patient has two X-ray images: cephalometric and OPG. Hence, the required action is to find the stage of the predicted molar by the predicted image of the cervical, and this process is used for both genders. Moreover, the vice-versa process is implemented as well, which means using the predicted cervical image to find the stage of molar class. So, there is a consideration of correlation between the two images after the prediction, which counts as 1 if there is a correlation between the two stages of growth and 0 if they are not correlated. As the implementation has been set on both genders, it is important to consider the different genders regarding the correlation and stage prediction for the two images.

The next step is that the image datasets in each model architecture are split into the train set and the validation set. The train sets are used to train the model and make it more general for unseen data. On the other hand, validation sets are used as unseen data to calculate the model’s accuracy before finalizing for production. So, the amount split is 20% for validation and the rest is for the training process, which is 80%. This process is implemented when the image datasets are categorized based on their class names into folders.

So, in the case of molar predications, there are considerations of six classes, which are from C to H, and they are the names of each folder. In the same way, the same process is implemented for cervical images, which are categorized into six classes named from CS1 to CS6, which means there are six folders. So, this action is used for both genders in separate folders, named females and males.

After the process of splitting the images into folders based on their categories, the next step is to design the AI model, which is done by using the CNN model. The model takes the images that are in a greyscale channel. And they are cropped to capture the interesting part, which is (C2, C3, and C4) of vertebrae X-rays, and (Stage 1 to Stage 6) in the case of cervical 7th molar images.

Subsequently, the produced images were converted into the image size of 250 by 250 for each image category, and this size helps to capture more features from images to be extracted during the CNN process. If the images are not in the same shape width, and height, this leads to inaccurate performance and low accuracy results.

Another important step is that the images go through a process, called downscaling, by using the min–max scaling technique, which works on image pixels and converts them into the same range between 0 and 1. This process is important, as the model works on many images, so that there are different ranges of pixel values in different regions of different input images. Also, following this process ensures increases in the performance of the model, rather than working on large pixel values.

In addition, to avoid overfitting cases, the images go through a process of augmentation. This process takes each image and creates other images of different shapes. The augmentation process in CNN involves transformation, scaling, flipping, translations, and rotations. At the end of this process, it can be assured that many images are provided, which decreases the amount of overfit and increases prediction accuracy.

Also, the process of augmentation is applied on a train set only; the reason is that the CNN model is required to train on more data and different shapes of images. The validation set is used for testing the model to show how the model performs on unseen data.

After that, the CNN model is created, and it has four layers of convolutions with different filter sizes, which are 3 by 3, including batch normalization and a dropout layer, which works on increasing the performance of the model and lessening the action of overfitting to be accurate.

This filter extracts the most important features from each image during the training process, such as edges and different relevant contents from images. Contents that can be used as features are most repeated or distinguished among other images.

In addition to the filtering process, a stride of one is used to shift one column at a time on each column of pixels in the images. Also, after each level of convolution, the max pooling technique is used to make the extracted feature more focusable and reduce the size of the image to only the part that is considered to be more relevant for predictions.

**Figure 3:**
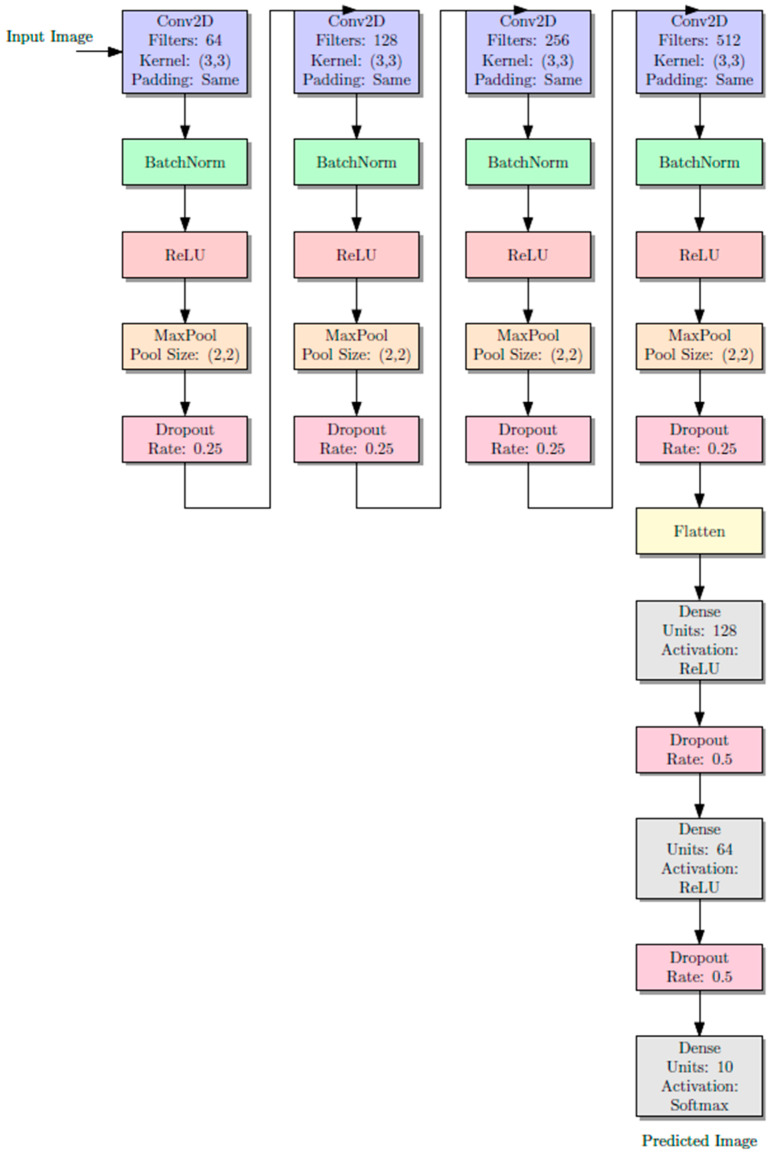
The architecture of CNN mode.(The purple color is for convolution layer, The light green color is for batch normalization layer, The light pink color is for Relu optimization layer, The light purple is for maxpooling layer, Pinkish red is for dropout layer, The pale yellow is for flatten, The final two light blue is for dense layers.

At the last step of the CNN process, the next level starts with flattening all the pixels of the generated features as a one-dimensional array. Then, it is passed into a two-level ANN (artificial neural network), with batch normalization of 0.3 after each level. In this step, the predicted output is resulted out. Figure 3 shows the model architecture of CNN. Also, it illustrates CNN’s designed architecture model, which is used for image classification efficiently. This process requires a set of layer combinations.

- Convolutional neural network works on extracting features using kernel filters 3 × 3 with conv2d sizes of 64, 128, 256, and 512 in consequences.
- Batch normalization to prevent gradient vanishing and accelerate the training process.
- Activation function, which works on extracting more complex patterns from the images, and rectified linear unit (ReLu), which is non-linear activation, is used as an activation function type.
- Max pooling works on reducing the spatial dimensionalities of the extracted feature maps, which decreases computational time and computer resources, and 2 × 2 max pooling size is used.
- The dropout layer forces the architecture network to learn more robust and generalized features, and 0.25 units are used during the convolution process, while 0.5 units are used during the flattening and fully connected layer, which are the last stage of the training model.
- Flatten layer converts the 2D feature into a 1D vector, and this flattening process prepares the data for the next layer, which is a fully connected layer.
- This fully connected layer works on learning the patterns and data of the images. In this stage, a higher-level representation is formed to make the final predictions on the input images.
- Output layer: This is the final layer of the model, and it uses the SoftMax function to convert the output to probability distributions. These distributions represent the likelihood that the input image belongs to a particular class, so the class with the highest probability is selected as the final prediction.

**Figure 4:**
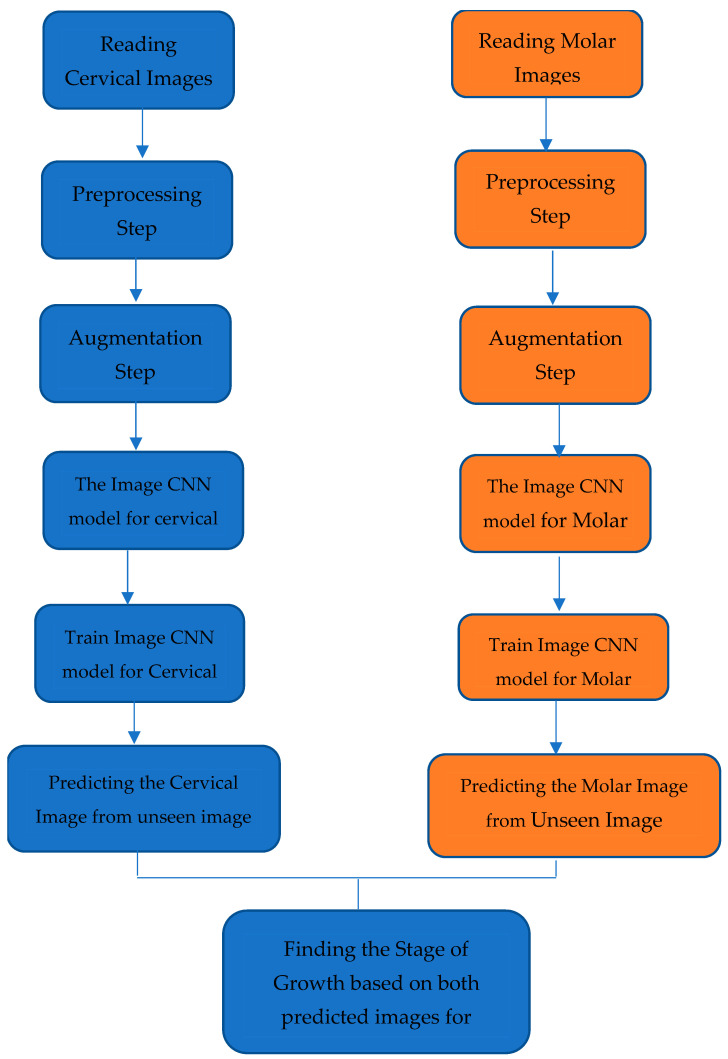
The architecture of CNN mode, the same model is implemented for both genders (the blue box for Cervical maturation index, orange box for Second molar calcification level).

Figure 4 illustrates data handling and model training strategies in our study. Also, this flowchart provides an overview of the steps taken to ensure the quality of the model and optimizations of model quality.

#### 2.3.1. Reading Images

The process starts with reading cervical and molar images, which are the primary input to the model for subsequent steps.

#### 2.3.2. Preprocessing Step

Both images undergo preprocessing, which involves resizing, normalization, and noise reduction. This step is also essential to standardizing the input data. The non-local means filter is used, which compares and averages similar pixel values throughout the image rather than just nearby ones, reducing noise while preserving details.

This noise reduction filter is implemented on all the image pixels with similar patches, not only the neighbor pixels. Then, a weighted average of the similar patches will be taken into account so that more similar patches will contribute to the final pixel value. As a result, the output will be much more effective with noise reduction, specifically for repetitive structure or texture in images. So that the model would be able to correspond to images that may have noises, and it would remove the noise and then pass it to the CNN model.

**Figure 5:**
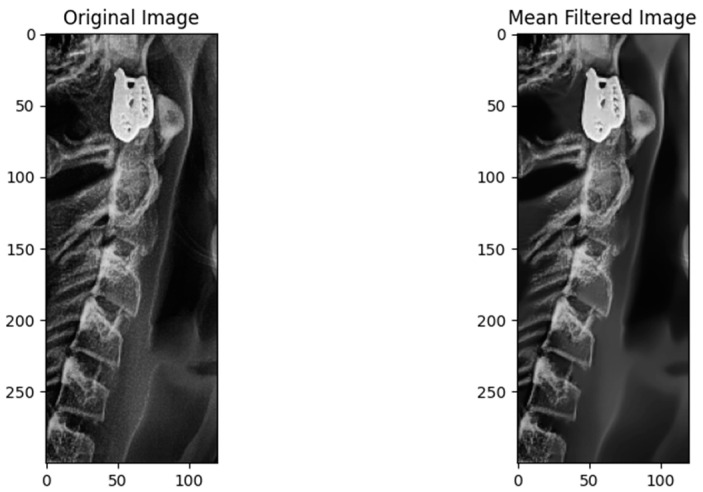
Applying non-local means filter on cervical.

**Figure 6:**
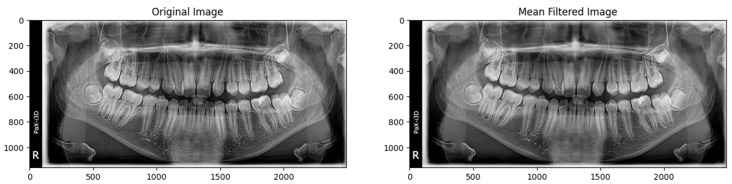
Applying non-local means filter on molar.

Some of the images in our dataset contain noises, which affect the accuracy of the model prediction, because these noises obscure important features such as edges and textures, losing some important information. After implementing the non-local means filter technique, it cleans all the images in our dataset, and keep all the relevant information. As the model works on extracting features (edges, shapes and textures) from the inserted images After removing the noises the accuracy of the model is increased, this because the model more focus on the relevant feature for detections, as shown in Figure 5 and Figure 6.

The noise reduction process starts after reading images from the image directory for both categories, the cervical and molar images. Each image is read and inserted into the non-local means noise reduction process. The output image is saved into the new directory, preparing for the next operation, which is image augmentation.

#### 2.3.3. Augmentation Step

After the preprocessing task, images are augmented to enhance the model’s training process. This step includes techniques like flipping, zooming, and rotation. This helps the trained model to become more generalized. These variations generate new training samples that are different from the original images and make the model learn patterns and features across different variations, and this improves robustness and ability to generalize to unseen images. with different variations and shapes.

The argumentation step is used because, without it, the model will train on a static number of images and does not expose it to different perspectives of the image dataset. Therefore, this can lead to overfitting, which is the case where the model trains only on a small number of image datasets; it means it performs well on training datasets and poorly on unseen images or images that it has not learned to its patterns and generalized it.

**Figure 7:**
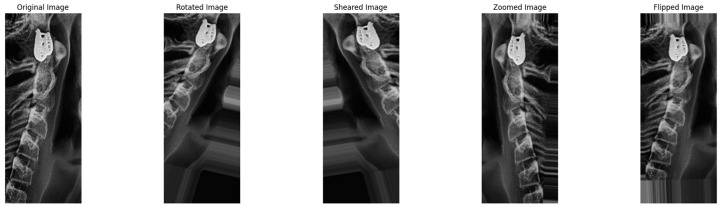
Implementing augmentation technique.

In addition, after implementing the argumentation step, the number of images in our datasets increases. Each image of the cervical and molar undergoes the process of flipping, zooming, shifting, and rotation. Thus, the model will train on different image orientations and sizes so it can be more generalizable to unseen data. For this case, a Python augmentation package named Image Data Generator is used, as shown in Figure 7.

Also, the operation of this step is started after the process of noise reduction is finalized, and then the set of images is implemented into the augmentation process. A list of new images is generated, and after that, they are fed into the CNN model for training purposes. In this way, the proposed model becomes more generalized to unseen and future data because it becomes able to find different patterns and features of the inserted images.

#### 2.3.4. CNN Model Architecture

The preprocessed and augmented images are fed into separate convolutional neural network (CNN) model architectures for both images cervical and molar images. This model works on extracting relevant features from the images.

##### Model Training Process

The CNN models are trained separately for both images. This training process optimizes the model to predict the image characteristics accurately, which is essential for growth stage analysis.

##### Prediction Process

After the training model is finalized, the models predict outcomes on unseen cervical and molar images. This is important for evaluating the performance of the model’s quality and accuracy.

##### Growth Stage Determination

The final stage includes the integration of prediction outcomes from both models to investigate the overall stage of growth based on the combined cervical and molar images.

In addition, the model followed a set of mathematical equations, which perform mathematical equations to compute the working model on predictions.

##### Convolution Operation Equation

$$Z_{i,j,k}=(X*W)k_{=}\sum_{m=1}^{M}\sum_{n=1}^{N}X_{i+m-1,\;j+n-1}\;.W_{m,n,k}+b_{k}$$

where W is the kernel filter applied on the input image X to produce feature map Z, the following is the detail of the CNN model equation.

$Z_{i,j,k}$: The output value of the feature map is at position (i,j) in the k-channel.
$X_{i+m-1,\;j+n-1}$: The input value at the corresponding position.
$W_{m,n,k}$: The weight of the filter at position (m,n) in the K-channel.
$b_{k}$: The bias term for the K-th channel.
∗: Convolution operation.

Activation function, rectified linear unit (ReLU) activation function:

f(x) = max(0, x)

where f(x): indicates the output function, which depends on input x values.

x: is the input to the function, where x is the output from the previous CNN layer and is used with ReLU.
max(0,x): this operation returns the maximum output of two values 0, x; if x is positive or zero, the function returns x; if x is negative, it returns 0.

SoftMax function:

$$\mathrm{SoftMax}(z{)}_{i}=\frac{e^{zi}}{\sum_{j=1}^{k}e^{zj}}$$

where $e^{zj}\;$ is the standard exponential function for the input vector.

$e^{zj}$: standard exponential function for output vector.
K: number of classes in the multi-class classifier.

Moreover, the hyperparameters used in the models were set as follows: learning rate = 0.0001, batch_size = 16, number of epochs = 100, optimizer is Adam, kernel size (3 × 3), dropout layers=0.5, and the number of filters in the first convolution layer is 64, followed by 128, then 256 and 512 in consequent layers.

The implementation of the CNN model was done by using Python 3.10.12, Tensorflow, and Keras 2.17.0. It is implemented on the Google Colab iCloud services, as they provide free GPU and memory.

#### 2.3.5. Implementation Details

I. 2nd molar image predictions

1. Initializing the directories and classes
  - Define the directories that hold the datasets for male and gender.
  - Define the class list: [C, D, E, F, G, H].
2. Loading dataset images from directories
  - Create two empty lists for holding image paths and image labels.
  - For each class name in classes, getting all image files for each class
  - Adding image paths and their labels to two separate array lists.
3. Splitting dataset into training and testing
  - Split all images and labels into train images, test images, train labels, and test labels using the train_test_split() method with 80% for training and 20% for testing.
4. Convert labels to categorical format
  - Convert train—labels and test—labels to one-hot encoding using to_categorical() function.
5. Handle class imbalance
  - Compute class_weigts using compute_class_weight() to handle imbalance classes.
6. Create a data generator using Data augmentation
  - Initialize image data generator with rescaling and light augmentation (zoom, shear, and brightness adjustment) using ImageDataGenerator().
  - Create generators for images to be implemented with the augmentation process.
7. Building CNN model
  - Define the sequential model.
  - Add a convolution layer with 32 filters and ReLU activation.
  - Add max pooling layer
  - Add batch normalization layer
  - Add a convolution layer with 64 filters and ReLU activation.
  - Add max pooling layer
  - Add batch normalization layer
  - Flatten the output
  - Add a dense layer with 256 units and ReLU activation.
  - Add a dropout layer to prevent overfitting
  - Add a final dense layer with SoftMax activation, which is used to classify the input into one of the 6 classes.
8. Compile the model
  - Compile the model with adam optimizer, categorical_crossentropy loss and accuracy as the metric.
9. Train the model
  - Train the model with the train_generator method for a fixed number of epochs, which is 20.
  - Validating the model with a validation_generator function.
10. Evaluate the model with test set
  - Create the test_generator using the same ImageDataGenerator.
  - Evaluate the model using the test set and printing the accuracy result.
11. Predict on test set
  - Get the true label from test_generator()
  - Use model.predict() to predict the probabilities for each image in the test set.
  - Use argmax() to convert the predicted probabilities to class labels.
12. calculate the performance metrics
  - Calculate the weighted F1-score using f1_score() based on true and predicted labels.

II. cervical image predictions

1. Initializing the directories and classes
  - Define the directories that hold the datasets for male and gender.
  - Define the class list: [CS1, CS2, CS3, CS4, CS5, CS6].
2. Loading dataset images from directories
  - Create two empty lists for holding image paths and image labels.
  - For each class name in classes, obtain all image files for each class
  - Adding image paths and their labels to two separate array lists.
3. Splitting dataset into training and testing
  - Split all images and labels into train images, test images, train labels, and test labels using the train_test_split() method with 80% for training and 20% for testing.
4. Convert labels to categorical format
  - Convert train—labels and test—labels to one-hot encoding using to_categorical() function.
5. Handle class imbalance
  - Compute class_weigts using compute_class_weight() to handle imbalance classes.
6. Create a data generator using data augmentation
  - Initialize Image Data Generator with rescaling and light augmentation (zoom, shear, and brightness adjustment) using ImageDataGenerator().
  - Create generators for images to be implemented with the augmentation process.
7. Building CNN model
  - Define the sequential model.
  - Add a convolution layer with 32 filters and ReLU activation.
  - Add max pooling layer
  - Add batch normalization layer
  - Add a convolution layer with 64 filters and ReLU activation.
  - Add max pooling layer
  - Add batch normalization layer
  - Add a convolution layer with 128 filters and ReLU activation.
  - Add max pooling layer
  - Add batch normalization layer
  - Flatten the output
  - Add a dense layer with 256 units and ReLU activation.
  - Add a dense layer with 128 units and ReLU activation.
  - Add a dropout layer to prevent overfitting
  - Add a final dense layer with SoftMax activation, which classifies the input into one of the 6 classes.
8. Compile the model
  - Compile the model with adam optimizer, categorical_crossentropy loss and accuracy as the metric.
9. Tra in the model
  - Train the model with the train_generator method for a fixed number of epochs, which is 20.
  - Validating the model with a validation_generator function.
10. Evaluate the model with test set
  - Create the test_generator using the same ImageDataGenerator.
  - Evaluate the model using the test set and printing the accuracy result.
11. Predict on test set
  - Get the true label from test_generator()
  - Use model.predict() to predict the probabilities for each image in the test set.
  - Use argmax() to convert the predicted probabilities to class labels.
12. calculate the performance metrics
  - Calculate the weighted F1-score using f1_score() based on true and predicted labels.

## 3. Result

The model’s final result demonstrates the high degree of accuracy with which each stage and gender can be predicted. Furthermore, both genders do well in picture classification—especially when it comes to medical images—with accuracy levels exceeding 95% for each category. The findings are broken down by gender and class in Table 1.

**Figure 8:**
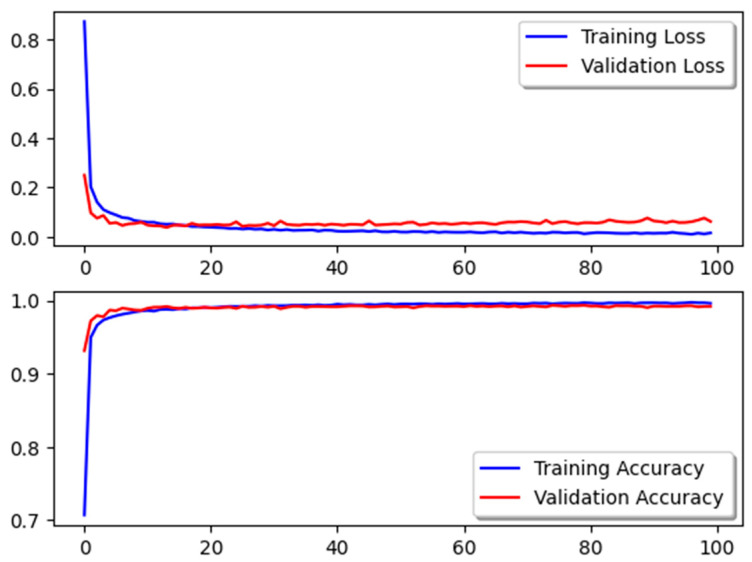
Accuracy and loss for training and testing for all CNN models.

Furthermore, the accuracy and validation rates demonstrate excellent performance and a discernible drop in loss. This indicates that there is a decreased likelihood of overfitting problems and incorrect predictions in the model as a whole. The accuracy of the model’s training performance is displayed in Figure 8, as well as the validation set and the decreasing loss amount.

In addition, the model is validated using previously unknown imaging data (blinded data), and additional pictures from the cervical and molar areas are included. The model accurately predicted the class for each of them, and it has been tested on both genders. Furthermore, it demonstrates that each cervical and molar class can be anticipated and that the projected cervical stage corresponds to a molar stage, as well as the association between cervical and molar. As a result, throughout the test, it was discovered that the stages of the female dataset were always one stage ahead of the male dataset, in both cervical and molar.

Another metric used to test the model’s efficiency is the F1-score, the harmonic mean of two essential metrics: precision and recall.

Prediction measures the accuracy of true positive predictions; for example, it calculates the proportion of true positives to the total number of positive predictions. Also, prediction tries to find how many actual positive instances are correctly predicted.

$$\mathrm{Precision}=\frac{True\;Positive\;(TP)}{True\;Positive\;\left(TP\right)+False\;Positive\;(FP)}$$

Whereas recall measures the model’s ability to identify all the actual positive instances, it tries to find the proportion of the true positive to the actual positive instances. This means recall focuses on how many positive instances the model correctly predicted.

$$\mathrm{Recall}=\frac{True\;Positive\;(TP)}{True\;Positive\;\left(TP\right)+False\;Negative\;(FN)}$$

The F1-score metric balances precision and recall to provide a single metric that calculates both false positives and negatives. The value of the F1-score ranges from 0 to 1, with the best value being 1 and the worst being 0. The results of the F1-score for our model are shown in Table 2.

$$F1\;\mathrm{Score}=2\;*\;\frac{Precision*Recall}{Precision+Recall}$$

In addition, another metric that showed the performance for the model were confusion matrixes, which are tables that evaluate the performance of a classification model. Also, it predicts the classes with the actual true classes, based on TP (true positive), FP (false positive), TN (true negative), and FN (false negative) for each class. The following Table 3 and Table 4 shows the performance metric evaluation using a confusion matrix for both cervical and 2nd molar image prediction accuracy.

## 4. Discussion

Dental examinations in modern dental clinics are supported by computer technologies that employ computational intelligence to detect potential health concerns more efficiently. Deep learning algorithms produce the most exact results in one of the most significant applications. A single-stage oriented deep learning model from scanning panoramic images for five dental treatments and diagnosis of teeth class revealed that the root canal treatment and impacted teeth had precision scores of 82.63% and 18.78%, respectively [20]. Furthermore, the TeethU2Net deep learning model used to detect tooth saliency by dental panoramic radiographs achieved a high accuracy of 0.97 [21].

In this paper, a unique correlation learning mechanism (CLM) for deep neural network topologies that blends CNN with traditional design was presented. The support neural network assists CNN in determining the best filers for pooling and convolution layers. As a result, the primary neural classifier learns more quickly and efficiently. The results reveal that our CLM model can achieve 96% accuracy, 95% precision, and 95% recall.

Skeletal growth prediction is critical in orthodontics and orthognathic surgery to arrange procedures corresponding to the patient’s development trajectory. It helps orthodontists to resolve skeletal abnormalities and optimize treatment outcomes properly. Regular exams and communication between orthodontists and other healthcare experts help to anticipate and treat skeletal development. The pubertal growth spurt is a key time of skeletal development throughout adolescence. It is critical for treatment planning to assess the time and amount of this growth surge. Skeletal growth prediction for dental implants is especially relevant when contemplating implant placement in developing persons or in places where continuing skeletal development may impair the implants’ long-term success.

Some specialized software programs are intended to forecast skeletal growth based on a variety of criteria. These instruments can help predict bone position in the future and guide implant placement.

Dental practitioners should think about long-term treatment plans for their patients, especially children. It might be done in stages, with interim remedies in place until skeletal growth stabilizes. The type of prosthetic repair chosen for the dental implant should take into account prospective skeletal expansion. This guarantees that the repair remains in sync with the evolving anatomy. It is critical to evaluate the implant site’s connection to surrounding tissues, such as sinuses or nerves. Skeletal development may have an effect on these interactions over time.

Predicting skeletal growth for dental implants requires an in-depth examination of the patient’s age, growth stage, and skeletal maturity, as well as modern imaging tools and coordination among several dental professionals. This method guarantees that dental implants are put in a way that allows for continuous bone growth while also maximizing long-term success.

Despite AI’s potentially revolutionary significance, the existing literature is lacking in recommended automatic solutions, despite some attempts in recent years.

Bone age is a measure of bone maturity that may be used to treat a variety of pediatric illnesses as well as legal difficulties. Traditional bone age evaluation is a sophisticated and time-consuming technique, prone to inter- and intra-observer variability, based on the study of distinct skeletal segments and teeth. Fully automated systems are in high demand, but developing an accurate and dependable solution has proven challenging. Deep learning technologies, machine learning, and convolutional neural network-based systems have demonstrated promising results in automated bone age assessment. We discuss the evolution of bone age estimation, its use, and conventional techniques of evaluation, as well as the current artificial intelligence-based solutions for bone age assessment and its prospects [22].

Ameli et al. (2023) analyzed the form and pattern of cervical vertebrae; machine learning models were used to 3D cephalometric pictures to forecast the development stage of patients [23]. However, the quantity of radiation exposure, which is more than that of a 2D cephalogram, is the source of the most debate concerning its use in dental imaging [24]. Although CBCT pictures are indicated to be a reliable and effective technique for assessing skeletal age using CVM, they should not be utilized primarily for that purpose [25].

Fifty-eight percent training and fifty-seven percent test accuracy were acquired as a result of the 40-epoch training. The model produced findings that were extremely close to those acquired during training on the test data. On the other hand, it was established that the model performed best in terms of accuracy and F1-score in CVM Stage 1 and best in terms of recall value in CVM Stage 2. According to the experimental data, the constructed model had reasonable success, with a classification accuracy of 58.66% in CVM stage classification [26].

Machine learning algorithms can assist in cephalometric analysis by automatically identifying landmarks on X-rays and measuring various parameters related to facial and dental structures. This can help orthodontists in diagnosing malocclusions and planning treatment [27]. A study used a customized open-source CNN deep learning algorithm analysis in comparing an automated cephalometric analysis to the experienced human examiner in determining 18 landmarks on a total of 1792 cephalometric X-rays, and they reported no statistically significant differences between humans’ gold standard and the AI’s predictions [28].

Classifying images is a complex problem in the field of computer vision. The deep learning algorithm is a computerized model that simulates human brain functions and operations. Training the deep learning model is costly in machine resources and time. Investigating the performance of the deep learning algorithm is mostly needed. The convolutional neural network (CNN) is most commonly used to build a structure of deep learning models. The final results evaluate the deep learning algorithm as a state-of-the-art method for an image classification task [29].

The CNN model is used to care for farming by identifying leaf diseases that help in growing up healthy plants [30], breast cancer abnormalities [31], skin cancer [32,33], lung disease [34], and so on. Still, this study is the first one dealing with growth prediction. A study by Rauf et al. (2023) recommends using K-nearest neighbor to predict arch perimeters rather than linear regression [35].

Two studies were conducted in the Iranian population to estimate dental age; one revealed that mandibular third molar calcification could be used as a dental age predictor, especially in males [13]. Another study indicated a high correlation between mandibular second molar calcification and skeletal maturity in the post-pubertal growth phase [36].

Relying on OPG and cephalometric radiographs is that these methods are widely available and cost-effective, making them accessible for a larger patient population. In many clinical settings, OPG and cephalometric radiographs are the standard imaging techniques, allowing for easier integration into routine dental and orthodontic practices. Additionally, the study may have aimed to establish foundational data using these established methods before potentially exploring more advanced 3D imaging techniques in future research. By starting with OPG and cephalometric radiographs, researchers can build a comprehensive dataset that could later be enhanced with 3D imaging for more nuanced analyses. Moreover, while 2D imaging may have limitations, it still provides valuable insights into skeletal relationships and developmental patterns, which can serve as a basis for understanding broader trends in skeletal maturation. In addition, CBCT cannot be recommended for every orthodontic patient unless there is an impacted tooth, surgical correction, or a pathological condition due to increased radiation dose, risks, safety, ethical, and medico-legal considerations.

Object detection is the most important problem in computer vision tasks; CNN (convolutional neural network) can perform well even with moderate-sized datasets. The reason is that convolution structures inherently capture local spatial relationships, which is particularly advantageous in image classification tasks. Also, CNN does not highly require having large-scale datasets and can perform well on smaller datasets as well. Unlike transformers, which require more data to perform well and learn effectively.

In terms of performance and computational efficiency, transformers such as the vison transformer (ViT) excel in large datasets but tend to have very computational demands. Whereas CNNs have been optimized to be lightweight and computationally efficient while maintaining strong accuracy, which is advantageous in real-world applications where resources are limited.

In addition, CNNs are best used for image classification, object detection in images, and image segmentation. While transformers are best used for NLP (natural language processing), text reorganization, and summarization. Also, transformers can be used for multimodal tasks such as video understanding and captioning.

Based on our medium-sized dataset used in this study and the priority to optimize the computational efficiency, CNNs were chosen over transformed based models. Also, CNNs are well suited for tasks requiring lower computational resources while maintaining high predictive accuracy on moderate datasets, which makes them an ideal choice.

CNNs make image problems easier to handle because of their transitional invariance and inductive bias features. The training model needs a lot more data or more robust data improvement to learn picture attributes because transformers lack this feature [37].

Accuracy is not a trustworthy metric on its own when working with skewed datasets. It may be deceptive since a model may perform poorly overall even if it consistently predicts the majority class correctly. The following significant metrics were taken into account: false positives (FP) are instances of the positive class that are incorrectly predicted, false negatives (FN) are instances of the negative class that are incorrectly predicted, true positives (TP) are instances of the positive class that are correctly predicted, and true negatives (TN) are instances of the negative class that are incorrectly predicted.

Precision, which gauges the precision of optimistic forecasts, is another derived metric. Recall (sensitivity) assesses the capacity to identify every positive example. The F1-score, which is helpful for balancing precision and memory, is the harmonic mean of the two. Confusion matrix is a table that makes it possible to see how well a classification model is performing. For machine learning initiatives to be successful, it is essential to grasp the appropriate evaluation criteria and strike a balance in class representation. The dangers of class imbalance can be lessened with careful consideration of the evaluation technique, algorithm choice, and data characteristics [38].

In this study, by providing a thorough evaluation of each preprocessing step and discussing their limitations and challenges, we can present a more balanced and comprehensive view. This will help readers understand not only the benefits but also the complexities involved in preprocessing for improved model performance. Furthermore, it demonstrates that each cervical and molar class can be anticipated and that the projected cervical stage corresponds to a molar stage, as well as the association between cervical and molar.

## 5. Conclusions

In this study, a unique correlation learning mechanism (CLM) for deep neural network topologies that blends CNN with traditional design was presented. The support neural network assists CNN in determining the best filers for pooling and convolution layers. The results reveal that our CLM model can achieve 96% accuracy, 95% precision, and 95% recall. So, the calcification level of the lower second molar is a reliable method to trust in the growth level, so the traditional OPG is enough. CNN multiclass classification is an accurate method to detect the level of maturation of dental patients seeking treatment, whether from cervical maturation or the calcification of the lower second molar.

## Figures and Tables

**Table 1:** The accuracy result per each class for each gender.

No.	Gender	Cervical Prediction Accuracy	Second Molar Prediction Accuracy
1.	Male	98%	96%
2.	Female	96%	97%

**Table 2:** The F1-score result per each class for each gender.

No.	Gender	Cervical F1-Score	Second Molar F1-Score
1.	Male	0.93	0.91
2.	Female	0.91	0.92

**Table 3:** Cervical image confusion matrix table for male and female.

	Predicted CS1	Predicted CS2	Predicted CS3	Predicted CS4	Predicted CS5	Predicted CS6
Actual CS1	199	1	0	0	0	0
Actual CS2	2	198	0	0	0	0
Actual CS3	0	0	199	1	0	0
Actual CS4	0	0	1	198	1	0
Actual CS5	0	0	0	1	197	2
Actual CS6	0	0	0	1	1	198

**Table 4:** Second molar image confusion matrix table for male and female.

	Predicted C	Predicted D	Predicted E	Predicted F	Predicted G	Predicted H
Actual C	196	2	1	1	0	0
Actual D	1	198	1	0	0	0
Actual E	1	1	197	1	0	0
Actual F	0	0	0	198	1	1
Actual G	0	0	0	0	199	1
Actual H	0	0	0	0	2	198

**Figure A1:**
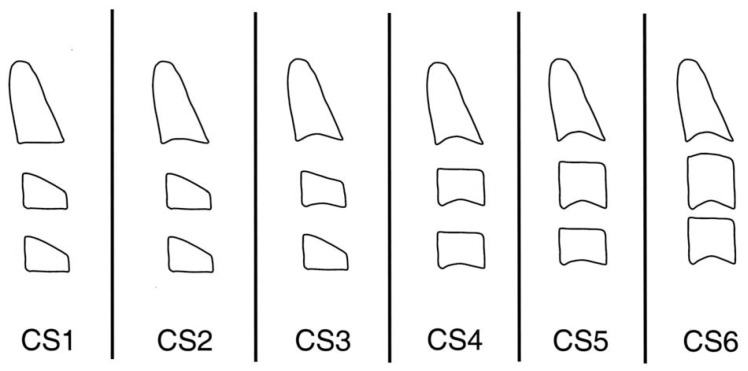
Schematic representation of the stages of cervical vertebrae according to the newly modified method (Vijayashree, et al.: Second molar calcificaon as an skeletal maturity indicator). Cited by: McNamara et al. 2005 [18].

**Figure A2:**
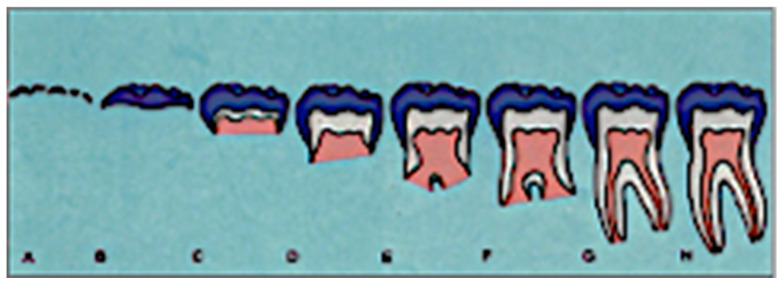
Developmental stages of tooth—Demirjian et al. (1973) [19]; color atlas of dental medicine; orthodontic diagnosis; Thomas grabber; 1992, Thieme Medical Publishers (page no. 100).

## Data Availability

The present study’s data are available with the corresponding author on request.

## Appendix B

The level of the growth captured from the amount of root calcification of the 2nd molar representing level E based on Demirjian et al. 1973 [19] as showed in the next figure.
